# Predictors of Fatal Outcomes among Pediatric Patients Hospitalized for Rocky Mountain Spotted Fever, Sonora, Mexico, 2004–2024^1^

**DOI:** 10.3201/eid3202.251223

**Published:** 2026-02

**Authors:** Stephanie Bellman, Kaci D. McCoy, Diana Enriquez, Pamela Romo, JongIn Hwang, Kathleen Weimer, Sarah M. Gunter, José Luis Alomía-Zegarra, Kristy O. Murray, Gerardo Álvarez-Hernández

**Affiliations:** Emory University School of Medicine, Atlanta, Georgia, USA (S. Bellman, K.D. McCoy, J. Hwang, K. Weimer, K.O. Murray); Children’s Healthcare of Atlanta, Atlanta (S. Bellman, K.D. McCoy, J. Hwang, K. Weimer, K.O. Murray); University of Sonora, Hermosillo, Mexico (D. Enriquez, P. Romo, G. Álvarez-Hernández); Baylor College of Medicine and Texas Children’s Hospital, Houston, Texas, USA (S.M. Gunter); Secretaría de Salud Pública del Estado de Sonora, Hermosillo (J.L. Alomía-Zegarra); Hospital Infantil del Estado de Sonora, Hermosillo (G. Álvarez-Hernández)

**Keywords:** Rocky Mountain spotted fever, Rickettsia, bacteria, vector-borne infections, vector-borne disease, tickborne illness, tickborne disease, pediatric, children, Mexico

## Abstract

The tickborne disease Rocky Mountain spotted fever (RMSF) remains life-threatening among children in northern Mexico. We retrospectively investigated 500 pediatric RMSF patients hospitalized in Sonora during 2004–2024. We analyzed clinical, laboratory, and sociodemographic data to identify predictors of fatality by using descriptive statistics and multivariable logistic regression. We found that the overall case-fatality rate was 19.8%, decreasing over time from 31.4% (2004–2013) to 14.5% (2014–2024). Fatal outcomes were associated with delayed doxycycline treatment (>5 days after symptom onset), older age, Indigenous background, and abnormal laboratory markers. Among survivors, 16% had life-altering sequelae, including amputations and neurologic deficits. Cases occurred year-round, predominantly in urban settings. Timely doxycycline administration remains a critical factor in reducing mortality rates. Vulnerable populations, including persons living in poverty, children >10 years of age, and Indigenous communities, require targeted interventions. Strengthening early diagnosis and understanding mechanisms underlying severe disease and death could improve RMSF outcomes in endemic regions.

Rocky Mountain spotted fever (RMSF) is a severe, often fatal tickborne disease caused by *Rickettsia rickettsii* bacteria (*1*). Although multiple hard tick species carry *R. rickettsii*, the brown dog tick (*Rhipicephalus sanguineus* sensu lato) has emerged as the predominant vector in the southwestern United States and in Mexico (*2*,*3*). RMSF is associated with high case-fatality rates (CFRs), particularly in communities with lower socioeconomic status where close contact with tick-infested dogs is common (*4*).

RMSF is endemic in the Americas (*1*). During 2009–2023, Mexico reported 9,153 cases of spotted fever rickettsioses (SFR), including RMSF, nearly half of which occurred in US-bordering states. The state of Sonora had the highest CFR of the region; 37.9% of patients died from their infections (*5*), exceeding the 24% CFRs in the United States in the preantibiotic era (*6*), and far exceeding current US CFR estimates of 5%–10% (*7*). Of note, in Sonora, children accounted for more than half of confirmed RMSF cases and more than one quarter of deaths (*5*).

Without prompt doxycycline treatment, RMSF can rapidly progress to multiorgan failure and death; survivors can experience life-altering sequelae, including neurologic deficits or limb amputations (*7*,*8*). Treatment delays of >5 days from symptom onset triple the risk for death (*8*). Early symptoms, including fever, malaise, headache, and rash, are nonspecific and resemble other diseases like dengue and COVID-19 (*9*,*10*). Diagnostic limitations further complicate timely treatment: indirect immunofluorescent antibody (IFA) testing often requires send-out testing and confirmation of paired acute and convalescent samples (*11*), which typically takes days to weeks; PCR lacks sensitivity (*12*); and immunohistochemical (IHC) staining is not widely available and requires collection of tissue, often a punch biopsy of a rash lesion (*13*).

Previous studies identified clinical predictors of fatal outcomes in children, including septic shock, acute kidney injury, neurologic complications, hemorrhages, and hemophagocytic lymphohistiocytosis (*8*,*14*,*15*). Treatment-related factors, such as mechanical ventilation, inotropic support, intravenous fluid supplementation, and delayed doxycycline administration, also contribute to adverse outcomes (*16*). Although those aspects have been researched in Sonora (*8*,*10*,*17*), how clinical manifestations, treatment, and outcomes of hospitalized pediatric patients have evolved over the past 2 decades remain unclear. We examined trends in pediatric RMSF case-patients hospitalized at Sonora’s main public pediatric hospital during 2004–2024 to describe clinical characteristics and identify predictors of severe and fatal outcomes that can inform strategies to improve outcomes for children infected with RMSF.

## Materials and Methods

### Data Collection

We conducted a retrospective analysis of medical records from all children hospitalized with suspected SFR at the primary public pediatric referral center in the state of Sonora, Hospital Infantil del Estado de Sonora (HIES), during January 2, 2004–December 31, 2024. Case-patients included those who had RMSF or SFR diagnoses and an acute illness of <2 weeks’ duration that was characterized by fever, headache or irritability, and a rash that might involve the palms and soles. We assigned patients codes A77.0 (Spotted fever due to *Rickettsia rickettsii*) or A77.9 (Spotted fever, unspecified) from the International Classification of Diseases, 10th Revision (ICD-10) (*18*), on the basis of final classification. Laboratory confirmation was established either through a single blood sample testing positive for *R. rickettsii* or *Rickettsia* spp. by PCR or by positive IgG titer >1:64 using IFA. When laboratory confirmation was not possible, we used clinical and epidemiologic criteria to support the diagnosis. We identified a total of 558 case-patients and excluded 58 case-patients for whom we were unable to confirm clinical, laboratory, or discharge diagnoses, yielding a total of 500 case-patients for analysis. Original data collection was approved by the Research Ethics Committee (registration no. 2869) of HIES (registration no. 003/23, approved June 20, 2023), and secondary analysis of de-identified data was determined to be nonhuman subjects research by the institutional review board of Emory University (Atlanta, GA, USA) on April 1, 2025.

HIES investigators trained in standardized chart review procedures and blinded to the study objectives extracted data from medical records. Extracted variables included clinical signs and symptoms, laboratory data at admission, sociodemographic variables, method of diagnostic confirmation, and factors related to medical care (e.g., day of symptom onset, day of doxycycline initiation), as well as discharge outcomes. The socioeconomic status (SES) variable represents an internal categorization by HIES, which primarily serves patients without any formal health insurance system affiliation, generally representing middle- and low-income populations. HIES classifies patients into 8 socioeconomic categories, which we dichotomized as having either sufficient or insufficient economic resources.

When available, we categorized patient weights and heights as moderately underweight if the weight-for-age was <2 SD below the median for sex and malnourished if body mass index (BMI) was <2 below SD the median for children >24 months of age or if weight-for-length was below the second percentile for children <24 months of age. We made those categorizations on the basis of World Health Organization International Growth Charts for children <24 months (*19*) and the US Centers for Disease Control and Prevention (CDC) Growth Charts for persons >24 months of age (*20*), as recommended by CDC (*21*). Because CDC charts report percentiles, we calculated the 2 SD threshold of weight by using the formula documented (*22*).

### Data Analysis

We estimated annual cumulative incidence and mortality rates per million pediatric population from 2004–2024. The denominator for those calculations was the pediatric population (children 0–18 years of age) covered by the Ministry of Health, representing ≈32% of the total population. We calculated CFRs by using the number of deaths as the numerator and the number of confirmed case-patients at HIES as the denominator. We plotted polynomial-smoothed temporal trends for those epidemiologic indicators and assessed statistical significance by using the coefficient of determination (R^2^) and 95% CIs.

For statistical analysis, we primarily stratified study participants by case outcome (i.e., fatal or nonfatal) and period of occurrence (2004–2013 vs. 2014–2024). We additionally performed subanalyses, assessing disease severity by stratifying case-patients with long-term sequelae or death versus those whose disease resolved without sequelae, and among survivors, comparing those with long-term sequelae versus those without.

We used R version 4.5.0 (The R Project for Statistical Computing, https://www.r-project.org) to perform descriptive statistical analysis to evaluate the clinical characteristics of the study population. We assessed laboratory values both as continuous and categorical variables, classified as normal or abnormal on the basis of age-specific reference ranges (*23*). We used Kruskal-Wallis analysis of variance, Fisher exact test, and Pearson χ^2^ test to compare differences between groups. We considered p<0.05 statistically significant for all predictors except clinical symptoms and laboratory values, for which we considered p<0.001 significant, applying a Bonferroni correction for multiple comparisons (*24*).

We conducted bivariable and multivariable logistic regression analyses to assess associations between predictors and fatality and developed a multivariable model to evaluate sociodemographic and treatment-associated factors. To decrease collinearity, we selected predictors by choosing 1 representative variable per concept by lowest p value in bivariable analysis or, if all nonsignificant, least manipulated (e.g., continuous instead of categorical age) and excluded variables with >10 missing values. We entered all candidate variables into the model and assessed collinearity by using car package version 3.1-3 (all variance inflation factor <5; https://CRAN.R-project.org/package=car), followed by backward selection using the stepAIC function from MASS package version 7.3-65 (https://CRAN.R-project.org/package=MASS) to obtain the final model with the lowest Akaike information criteria (AIC). We also calculated adjusted odds ratios (aORs) and 95% CIs.

## Results

**Figure 1:**
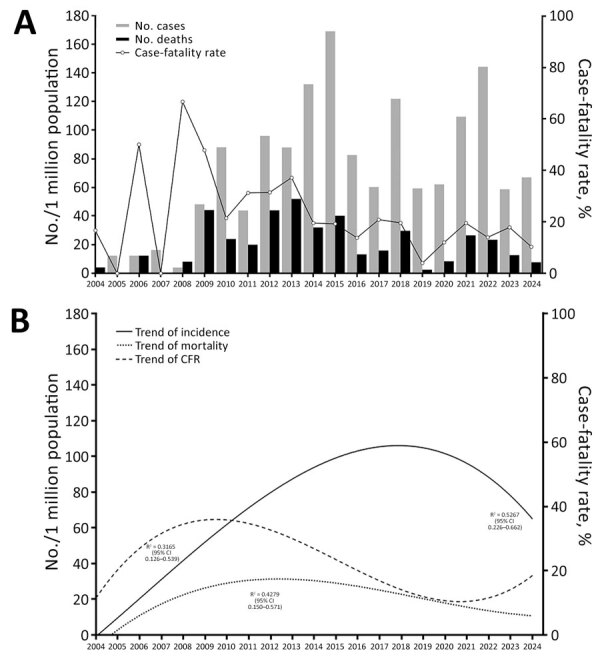
Incidence of cases and deaths per 1 million children and case-fatality rates over time in study of predictors of fatal outcomes among pediatric patients hospitalized for Rocky Mountain spotted fever, Sonora, Mexico, 2004–2024. A) Cumulative rates; B) polynomic trends. Scale bars for y-axes differ substantially to underscore patterns but do not permit direct comparisons.

RMSF incidence at HIES rose steadily during 2004–2015, then fluctuated, with subsequent peaks in 2018 and 2022 (Figure 1, panel A). During the study period, 500 children were hospitalized with RMSF, and 372 (74.4%) had laboratory-confirmed RMSF. The overall CFR was 19.8% (99/500), decreasing from 31.4% (49/156) in 2004–2013 to 14.5% (50/344) in 2014–2024 (odds ratio [OR] 0.37 [95% CI 0.24–0.58]; p<0.001). CFR rose until the early 2010s, then declined modestly (R^2^ = 0.3326), with a relative increase after 2020. Mortality rates began to decline around 2013 and then stabilized at ≈20 deaths/1 million children served by the Ministry of Health annually (Figure 1, panel B).

In addition to the high mortality rates, 16% (n = 64) of survivors experienced severe, permanent sequelae, including amputations and neurologic impairment. Those outcomes declined from 25.3% (25/99) during 2004–2013 to 13% (39/301) during 2014–2024 (OR 0.50 [95% CI 0.29–0.89]; p = 0.016). Overall, 32.7% (163/499) of case-patients experienced either death or life-altering sequelae, a rate that nearly halved from 47.4% (74/156) during 2004–2013 to 25.9% (89/343) during 2014–2024.

**Figure 2:**
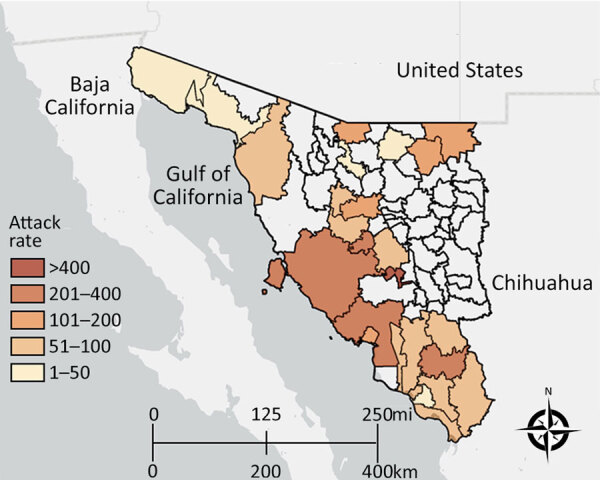
Attack rates by municipality of residence in study of predictors of fatal outcomes among pediatric patients hospitalized for Rocky Mountain spotted fever, Sonora, Mexico, 2004–2024. Map created by Esri ArcGIS Online (https://www.esri.com), June 2025. The map includes data from Esri, TomTom (https://www.tomtom.com), Garmin (https://www.garmin.com), Food and Agriculture Organization of the United Nations (https://www.fao.org), National Oceanic and Atmospheric Administration (https://www.noaa.gov), US Geological Survey (https://www.usgs.gov), OpenStreetMap contributors (https://www.openstreetmap.org), and the GIS User Community (https://communitymaps.arcgis.com).

Case-patients were concentrated in central and southern Sonora, particularly in Hermosillo, the state capital and most populous city (Figure 2). We noted an urban predominance; 420 case-patients were from urban areas versus 80 from rural regions; however, fatality rates did not differ significantly by setting (p = 0.36) (Table 1). RMSF cases occurred year-round, and we did not note any seasonal or monthly patterns (Appendix Figure). In addition, most (93.5%) children had a documented history of tick exposure before hospitalization.

**Table 1:** Sociodemographic characteristics of children in study of predictors of fatal outcomes among pediatric patients hospitalized for Rocky Mountain spotted fever, Sonora, Mexico, 2004–2024*

Characteristics	Fatal cases	Nonfatal cases	Total	p value†
Sex			500	
M	50 (50.5)	219 (54.6)	269 (53.8)	0.50
F	49 (49.5)	182 (45.4)	231 (46.2)	
Age range, y			500	
0–4	26 (26.3)	86 (21.4)	112 (22.4)	0.41
5–9	34 (34.3)	167 (41.7)	201 (40.2)	
10–14	27 (27.3)	113 (28.2)	140 (28.0)	
15–19	12 (12.1)	35 (8.7)	47 (9.4)	
Median age, y (IQR)	8.3 (4.1–12.3)	8.3 (5.3–11.6)	8.3 (5.3–11.8)	0.72
Residence			500	
Urban	80 (80.8)	340 (84.8)	420 (84.0)	0.36
Rural	19 (19.2)	61 (15.2)	80 (16.0)	
History of tick contact			492	
Y	86 (91.5)	374 (94.0)	460 (93.5)	0.36
N	8 (8.5)	24 (6.0)	32 (6.5)	
Socioeconomic status‡			499	
Insufficient	82 (82.8)	350 (87.5)	432 (86.6)	0.25
Sufficient	17 (17.2)	50 (12.5)	67 (13.4)	
Ethnicity			500	
Non-Indigenous	83 (83.8)	366 (91.3)	449 (89.8)	**0.04**
Indigenous	16 (16.2)	35 (8.7)	51 (10.2)	
Weight			260	
Underweight	3 (8.3)	17 (7.6)	20 (7.7)	0.75
BMI or weight-for-length§			260	
Malnourished	3 (3.8)	16 (7.1)	19 (7.3)	0.73

*Values are no. (%) except as indicated. Bold font indicates statistical significance (p<0.05). BMI, body mass index; IQR, interquartile range. †Based on Fisher exact test, Pearson’s χ^2^, or Kruskal-Wallis analysis of variance. ‡Hospital Infantil del Estado de Sonora classifies patients into 8 socioeconomic categories, which we dichotomized as having either sufficient or insufficient economic resources. §BMI was calculated for children >24 months of age; weight-for-length was calculated for children <24 months of age.

### Sociodemographic Features

Slightly more case-patients were male (54%) than female (46%); we found no significant association between sex and fatality (p = 0.50) (Table 1). Most (87%) children were classified as having insufficient SES; ≈10% were of Indigenous origin, representing 10 ethnic groups, and most resided in impoverished suburban or rural communities. During 2014–2024, older age was significantly associated with higher CFR, and the median age among fatal cases was 10.3 (interquartile range [IQR] 6.9–14.2) years versus 8.5 (IQR 5.5–12.2) years in nonfatal cases (p = 0.048); that association was not observed across the full study period.

### Clinical and Laboratory Features

**Figure 3:**
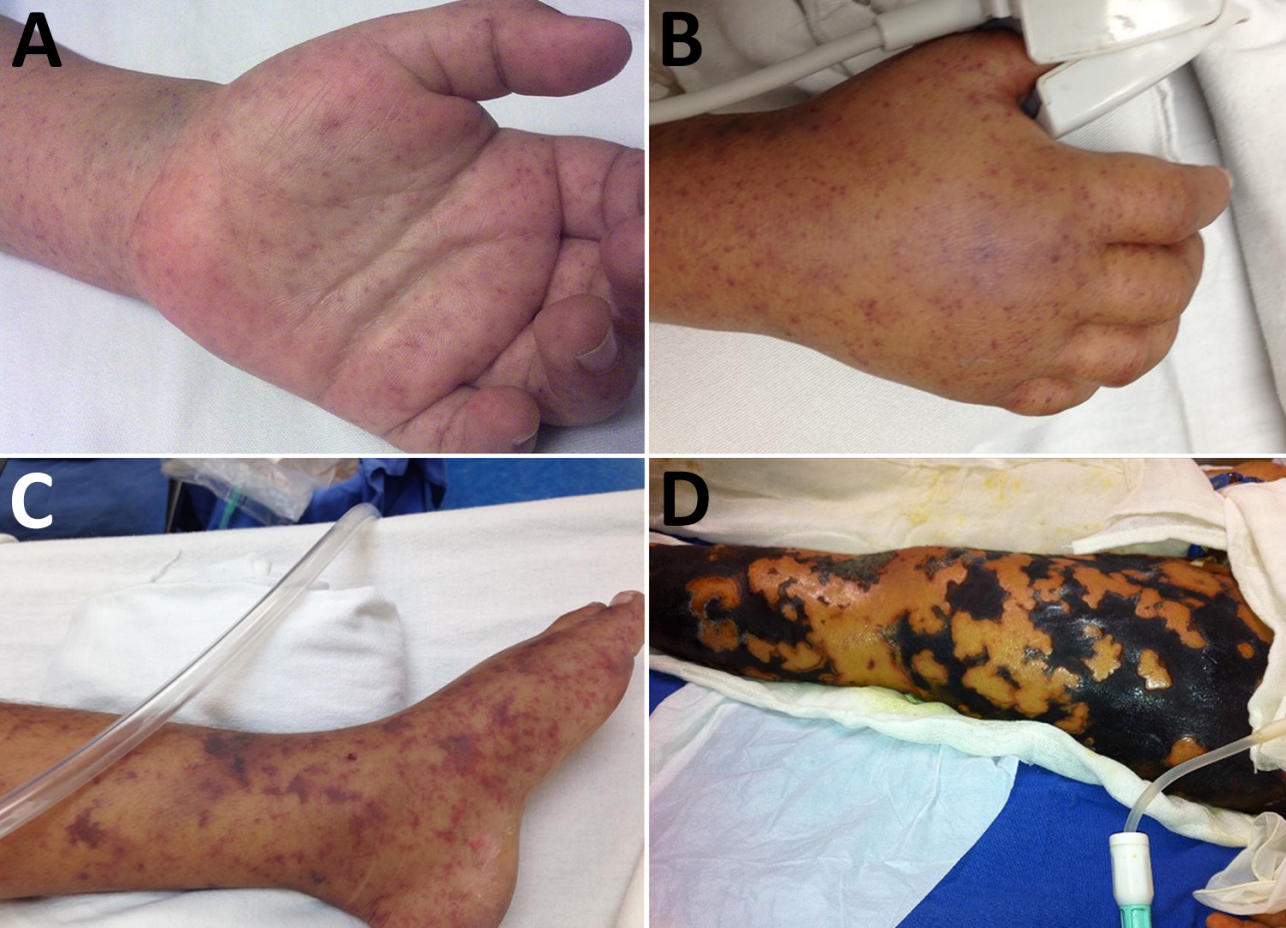
Clinical progress of children in in study of predictors of fatal outcomes among pediatric patients hospitalized for Rocky Mountain spotted fever, Sonora, Mexico, 2004–2024. A) Maculopapular rash on the palm of an 8-year-old child, observed on day 3 after symptom onset. B) Edema with petechia on the dorsum of the hand of a 6-year-old child, observed on day 5 after symptom onset. C) Ecchymotic patches on the foot and ankle of a 7-year-old child, observed on day 7 after symptom onset. D) Necrosis of the left lower limb in another 7-year-old child, observed on day 24 of disease progression.

The most common clinical features at hospital admission were fever (100%); exanthema (95%) (Figure 3, panels A, B), notably on palms (81%) or soles (77%); and headache (88%) (Appendix Table 1). Features significantly (p<0.001 for all) associated with fatal outcomes were vomiting, petechial rash, hemorrhage, ecchymosis and necrosis (Figure 3, panels C, D), respiratory distress, pulmonary and peripheral edema, hepatomegaly, neurologic alterations (e.g., altered mental status, confusion, seizures, encephalitis, coma), hypovolemia, and shock (Appendix Table 1).

The most frequent abnormal laboratory findings, defined as values outside age-specific reference ranges (*23*), were procalcitonin (98%), aspartate aminotransferase (AST) (92%), lactate dehydrogenase (LDH) (91%), platelet count (85%), prothrombin time (PT) (85%), alanine aminotransferase (ALT) (81%), and serum sodium (81%) (Appendix Table 2). Nearly all laboratory parameters we assessed were significantly associated with fatality (p<0.001), including leukocytosis, neutrophilia, elevated neutrophil-to-lymphocyte ratio (NLR), thrombocytopenia, elevated procalcitonin, prolonged prothrombin time (PT) and partial thromboplastin time (PTT), elevated liver enzymes (AST, ALT, LDH), elevated creatinine, low albumin, and low total protein (Table 2). Although both fatal and nonfatal cases had many abnormal values, those values were markedly more severe in the fatal group; for example, the median platelet count was 17,000/µL in fatal cases versus 60,000/µL in nonfatal cases (reference range 150,000–450,000/µL) (Table 2).

**Table 2:** Laboratory findings at admission for fatal and nonfatal cases in study of predictors of fatal outcomes among pediatric patients hospitalized for Rocky Mountain spotted fever, Sonora, Mexico, 2004–2024*

Indicator	No. cases	Median (interquartile range)			p value†
		Fatal	Nonfatal	Total	
Hemoglobin, g/dL	497	11.3 (10.3–12.8)	11.5 (10.3–12.5)	11.4 (10.3–12.5)	0.75
Hematocrit, %	494	34.1 (30.4–37.5)	33.7 (30.4–36.9)	33.8 (30.4–37.0)	0.92
Blood leukocytes, × 10^3^ cells/μL	498	17 (10–26)	8 (6–13)	9 (6–15)	**<0.001**
Absolute lymphocyte count, × 10^3^ cells/μL	475	1.8 (0.8–3.3)	1.1 (0.8–2.0)	1.2 (0.8–2.1)	0.007
Absolute neutrophil count, × 10^3^ cells/μL	477	11.1 (8.1–18.8)	5.9 (4.0–9.0)	6.7 (4.3–10.5)	**<0.001**
Neutrophil-to-lymphocyte ratio	471	8 (4–15)	5 (3–9)	5 (3–9)	**<0.001**
Platelets, × 10^3^ μL	497	17 (10–25)	60 (27–120)	46 (20–103)	**<0.001**
Serum procalcitonin, ng/mL	337	20 (8–44)	4 (1–11)	6 (2–18)	**<0.001**
Prothrombin time, sec	488	18.0 (15.8–20.7)	15.1 (13.9–16.2)	15.5 (14.1–17.1)	**<0.001**
Partial thromboplastin time, sec	486	47 (41–56)	39 (32–43)	40 (33–45)	**<0.001**
Aspartate aminotransferase, U/L	488	253 (171–398)	115 (61–165)	132 (70–205)	**<0.001**
Alanine aminotransferase, U/L	487	83 (65–115)	55 (34–82)	61 (37–90)	**<0.001**
Lactate dehydrogenase, U/L	384	1,479 (1,055–1,928)	752 (549–998)	839 (574–1224)	**<0.001**
Serum sodium level, mq/L	492	130 (126–135)	132 (129–136)	132 (129–136)	0.003
Serum creatinine, mg/dL	451	1.5 (0.8–2.6)	0.5 (0.4–0.6)	0.6 (0.4–0.9)	**<0.001**
Serum albumin, g/dL	416	2.8 (2.4–3.1)	3.2 (2.8–3.9)	3.1 (2.7–3.8)	**<0.001**
Serum total protein, mg/dL	417	5.0 (4.3–5.8)	5.8 (5.0–6.4)	5.6 (4.9–6.3)	**<0.001**

*Bold font indicates statistical significance (p<0.001). †Based on Kruskall-Wallis analysis of variance or Fisher exact test; statistical significance determined by Bonferroni correction.

### Clinical Course and Outcomes

**Figure 4:**
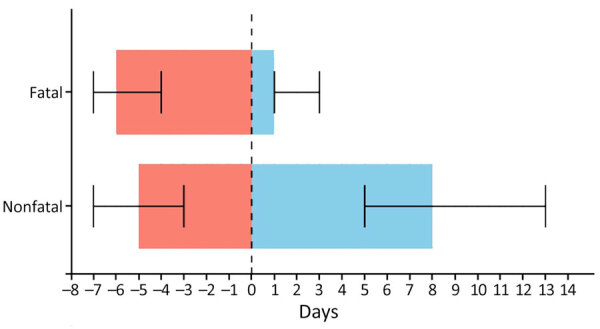
Analysis of days from symptom onset to hospitalization and length of hospital stay as predictors of fatal outcomes among pediatric patients hospitalized for Rocky Mountain spotted fever, Sonora, Mexico, 2004–2024. Horizontal bars represent median time from symptom onset to hospitalization (red bars) and length of hospital stay (blue bars) for fatal and nonfatal cases; whiskers indicate 95% CIs. Vertical dotted line indicates day of hospital admission.

Children with fatal outcomes were hospitalized later, at a median of 6 (IQR 4–7) days after symptom onset, compared with 5 (IQR 3–7) days in nonfatal cases (p<0.001) (Figure 4; Appendix Table 1). Children with later hospitalization times were also significantly more likely to develop complications during hospitalization, including vascular instability, secondary bacterial infections, neurologic disturbances, respiratory distress, acute renal failure, and shock (all p<0.001) (Table 3).

**Table 3:** Treatment and outcomes for fatal and nonfatal cases in study of predictors of fatal outcomes among pediatric patients hospitalized for Rocky Mountain spotted fever, Sonora, Mexico, 2004–2024*

Characteristic	No. cases	No. (%) cases			p value†
		Fatal	Nonfatal cases	Total	
Doxycycline initiated	500				**0.01**
<5 days		40 (40.4)	236 (58.9)	276 (55.2)	
>6 days		50 (50.5)	164 (40.9)	214 (42.8)	
Not treated		9 (9.1)	1 (0.2)	10 (2.0)	
Clinical complications‡					
Vascular§	496	80 (83.3)	106 (26.5)	186 (37.5)	**<0.001**
Bacterial¶	494	72 (75.0)	141 (35.4)	213 (43.1)	**<0.001**
Neurologic#	461	14 (21.5)	58 (14.6)	72 (15.6)	0.19
Acute respiratory distress syndrome	500	63 (63.6)	89 (22.2)	152 (30.4)	**<0.001**
Acute renal failure	499	60 (61.2)	27 (6.7)	87 (17.4)	**<0.001**
Shock	500	79 (79.8)	135 (33.7)	214 (42.8)	**<0.001**

*Bold font indicates statistical significance (p<0.05). †Based on Fisher exact or Pearson χ^2^ test. ‡Patients could have >1 complication. §Including hemorrhage, disseminated intravascular coagulation, necrosis, edema of target organs (brain, lung), and others. ¶Including nosocomial pneumonia, meningitis/encephalitis, septic hepatitis, bacterial endocarditis, and others. #Including neuromuscular disturbances, neuropsychiatric alterations, speech disorders, cerebral palsy, hepatic encephalopathy, and others.

Doxycycline was administered at a median of 6 (IQR 5–7) days of illness in fatal cases versus 5 (IQR 3–7) days in nonfatal cases. Delayed treatment (>5 days) was significantly associated with death (p = 0.01) (Table 3). During 2014–2024, physicians more frequently initiated doxycycline within 5 days of symptom onset than during 2004–2013 (59% vs. 50%). Overall, 98% (490/500) of children received doxycycline during hospitalization, and 55% (276/500) received it within 5 days of symptom onset. Of the 10 children who did not receive doxycycline, 9 died and 1 survived; 2 of those children (both with fatal outcomes) were from Indigenous backgrounds, and the other 8 children (7 fatal with outcomes) were non-Indigenous. Of note, 40 children died despite receiving timely treatment, representing 40% of recorded deaths.

### Multivariable Model

For the multivariable model, we assessed the following variables as potential predictors for death: sex, age, urbanicity, region, exposure to ticks, SES, Indigenous background, season (fall/winter/summer/spring), days of in-hospital treatment with doxycycline, time to doxycycline treatment, number of symptomatic days before hospital arrival, treatment with doxycycline monotherapy, and multidrug therapy. The best-fit model included ethnicity, days of in-hospital doxycycline treatment, time to doxycycline treatment, and doxycycline monotherapy (Table 4). After adjusting for other predictors, both a higher number of days from symptom onset to doxycycline treatment (aOR 1.19 [95% CI 1.08–1.31]) and Indigenous background (aOR 2.84 [95% CI 1.15–6.90]) were associated with higher odds of death. In contrast, doxycycline monotherapy (intravenous or oral) was protective (aOR 0.18 [95% CI 0.08–0.38]), as was more days of in-hospital doxycycline (aOR 0.63 [95% CI 0.57–0.69]) (Table 4).

**Table 4:** Sociodemographic and treatment predictors of fatal outcomes among pediatric patients hospitalized for Rocky Mountain spotted fever, Sonora, Mexico, 2004–2024*

Characteristic	β coefficient	SE	aOR (95% CI)†
Intercept	0.13	0.35	1.14 (0.58–2.25)
Ethnicity‡	1.04	0.45	2.84 (1.15–6.90)
Days of in-hospital doxycycline treatment	−0.46	0.05	0.63 (0.57–0.69)
Days from symptom onset to doxycycline treatment	0.17	0.05	1.19 (1.08–1.31)
Treatment with IV or oral doxycycline monotherapy§	−1.71	0.40	0.18 (0.08–0.38)

*Results of logistic multivariable analysis. aOR, adjusted odds ratio; IV, intravenous. †Accounting for all the variables included in the final model. Ten observations excluded due to missing data. ‡Referent was non-Indigenous. §Referent was no dose of doxycycline or inclusion of other antibiotics in treatment course.

### Morbidity Subanalysis

Among hospitalized RMSF survivors, 64 (16%) were discharged with long-term sequelae, including neurologic impairments, amputations, and cardiac or respiratory complications. Children with long-term sequalae were more likely to be Indigenous (p = 0.006) and have delayed doxycycline treatment (p = 0.04) than children discharged without sequelae. Children with long-term sequalae also more frequently had rash on the palms or soles, petechiae, and signs of disease progression (e.g., hepatomegaly, neurologic signs, edema, and shock) (Appendix Table 3). Laboratory abnormalities were also more common in that group, and we noted statistically significant differences in platelet count, AST, LDH, serum albumin, and total protein levels (all p<0.001) (Appendix Table 4). As in fatal cases, children who survived with sequelae had a median time to doxycycline treatment of 6 days, but children without sequalae were treated at a median of 5 days (Appendix Table 3). Analyses combining sequelae and death as adverse outcomes yielded results similar to those observed when comparing fatal to nonfatal cases.

## Discussion

Our findings indicate that RMSF remains a major pediatric health concern among the population in Sonora, Mexico. Although the CFR declined to 14.5% during 2014–2024, it remains twice as high as that of neighboring Arizona, USA (7%), during 2008–2017 (*25*). However, CFR was lower than the 24.1% observed in Brazil (2007–2015) (*26*) and CFRs from other areas of Mexico, ranging from 20.2% (*8*) to 58.8% (*27*). In those regions, fatal outcomes are closely linked to a complex interplay of social and ecologic determinants, including limited access to healthcare (*28*) and high exposure to *R. sanguineus*–infected ticks, particularly among children living near large populations of free-roaming or stray dogs (*29*).

We found that one third of children in this study died or experienced long-term disability because of RMSF. That finding highlights the need to systematically incorporate severe sequelae into clinical assessments to more accurately estimate the true burden of RMSF in pediatric populations and to avoid underestimation when only considering in-hospital deaths (*30*). Our analysis also revealed a shift in the age-related risk for death and that children >10 years of age had increased fatality rates during the last decade of the study period. Historically, children <10 years of age were considered the most vulnerable to severe RMSF outcomes (*31*). Although the reasons for the shift remain unclear, increased awareness and clinician training, particularly regarding doxycycline safety in young children, might partially explain the trend. Further research is needed to explore specific vulnerabilities to RMSF among adolescents.

We also found significant associations between Indigenous background and negative outcomes, including both death and long-term sequelae. That finding could partially be related to lack of doxycycline administration among the population because a greater percentage of Indigenous versus non-Indigenous children (3.9% vs. 1.8%) received no doxycycline treatment. That disparity also could reflect a range of factors not fully captured in our dataset. Although Indigenous populations in Mexico have historically faced systemic inequities in health access and social services (*32*), further research should explore additional contributors to poor outcomes in that population, such as the regional presence of highly virulent *R. rickettsii* genetic clades (e.g., Taiaçu and Sheila Smith) (*33*), glucose-6-phosphate dehydrogenase deficiency (*34*), barriers within primary or nonreferral healthcare systems, and social determinants related to language, beliefs, and cultural practices (*3*,*28*,*35*). Those factors are particularly relevant given the annual migration of ≈160,000 agricultural workers, many from diverse Indigenous communities, from southern to northwest Mexico (*36*), including RMSF-endemic areas of Sonora.

Although 53% of RMSF cases occurred during July–October, Sonora’s warmest months, we observed no clear seasonal pattern in incidence or mortality rates, consistent with previous reports (*37*,*38*). Therefore, clinicians should maintain a high index of suspicion for RMSF year-round to ensure timely diagnosis and treatment (*39*). Furthermore, our findings indicate that RMSF in this pediatric population primarily occurs in urban foci, contrasting with the historically rural and suburban distribution of the disease (*40*,*41*). In both the southwestern United States and in Mexico, RMSF is strongly associated with large populations of tick-infested stray and domestic dogs, often concentrated in densely populated, low-income areas, thereby enabling exposure and transmission (*29*,*42*).

The most frequently observed clinical symptoms (fever, rash, and headache) were highly nonspecific, underscoring challenges clinicians face in establishing timely diagnosis and treatment. That finding highlights the urgent need for rapid and reliable diagnostic tools to support early clinical decision-making and prevent disease progression. Currently available methods for confirming RMSF, such as IFA, PCR, and IHC, are valuable for epidemiologic purposes but have limited clinical efficacy because of methodologic constraints and the need for specialized personnel and infrastructure (*43*,*44*). Therefore, strengthening laboratory capacity and developing new rapid diagnostics are essential not only for enhancing RMSF surveillance but also to guide clinical decision-making and improve outcomes in resource-limited settings (*5*,*7*,*28*).

We found that symptoms associated with fatal RMSF outcomes were predominantly related to disruptions in coagulation, vascular permeability, and neurologic function. Those clinical manifestations were supported by abnormal laboratory findings, such as thrombocytopenia, hyponatremia, and elevated liver enzymes and creatinine, consistent with previous studies (*8*,*16*,*27*,*45*). Although many laboratory abnormalities were noted in all case-patients, elevated neutrophil counts, increased NLR (*46*), prolonged PTT, elevated creatinine, hypoalbuminemia, and low total protein were significantly more frequent and severe in fatal cases (p<0.001) (Table 2; Appendix Table 2). Although recognized in other disease contexts (*47*), some of those biomarkers have not been consistently emphasized in the RMSF literature, suggesting their potential as underrecognized indicators of severe and fatal outcomes.

Comparing the clinical manifestations of fatal versus nonfatal cases and survivors with sequalae versus those who fully recovered, several notable trends emerged. As expected, laboratory abnormalities increased with disease severity. Platelet counts were markedly lower in fatal cases (median 17,000/µL) and in survivors with sequelae (median 22,000/µL) compared with fully recovered survivors (median 72,000/µL) (reference range 150,000–450,000/µL), suggesting platelet count could serve as a key marker of RMSF severity. Children discharged with sequelae and those who died also experienced significantly more severe symptoms and complications (e.g., edema, hemorrhage, shock) during hospitalization (p<0.001). Of note, case-patients who died more often experienced vomiting and showed substantial differences in PT, PTT, and NLR than those who survived, patterns not observed when comparing surviving case-patients with and without sequalae. Those findings could aid in clinical stratification of disease severity and identifying predictors of fatal outcomes.

Most of the statistically significant clinical signs we identified reflect the severity and rapid progression of RMSF, typically emerging after the fifth day of illness. That delayed clinical manifestation hindered timely intervention and increased the risk for fatal outcomes (*48*), underscoring the need for predictive tools to identify high-risk patients earlier. We propose developing a composite clinical severity score or a machine learning–based algorithm integrating early clinical indicators (e.g., vital signs, basic laboratory parameters, epidemiologic risk factors) to stratify patients at initial examination. Those tools should be built using retrospective clinical data and prospectively validated to ensure rigor and ethical application. Their implementation in endemic areas could substantially improve early diagnosis, guide timely treatment, and reduce RMSF-related fatality.

Our findings reinforce that early doxycycline initiation is critical for avoiding severe outcomes; we found administration beyond 5 days after symptom onset was a strong predictor of death, consistent with previous reports (*3*,*8*). That finding is particularly concerning because during the first decade of our study, half of case-patients received doxycycline after the 5-day threshold. Although the percentage receiving doxycycline >5 days after symptom onset declined to 41% during 2014–2024, delays remained common. Educational efforts targeting healthcare providers and community members appear to have improved timely doxycycline administration, especially in younger children (*39*); nevertheless, further action is needed to improve early recognition and treatment across all ages. Timeliness was also limited by delayed hospital arrival; median time from symptom onset to hospital arrival was 5 days, leaving a narrow window for intervention. Although whether case-patients sought care or received treatment before HIES admission is unclear, the need for 2 key strategies is evident: increasing awareness among primary care providers to initiate doxycycline earlier or refer patients to hospital care without delay, and implementing community-based programs encouraging caregivers to seek immediate care for children with suspected RMSF (*5*,*28*).

The first limitation of this retrospective study is the possibility for selection bias because the cohort included only patients treated at HIES, which primarily treats uninsured and severely ill patients, potentially limiting generalizability to the broader pediatric population affected by RMSF in Sonora. Second, information bias is also a concern because of variability in medical record quality and absence of prehospital care data, and because 25.5% (128/500) of case-patients were clinically diagnosed without laboratory confirmation. As described previously, laboratory confirmation is a pervasive problem for SFR globally, especially in resource-limited settings. Although laboratory confirmation is preferred to validate SFR cases (*49*), clinical manifestations of our identified case-patients aligned with studies published by CDC (*2*,*7*), supporting our conclusions of SFR diagnosis. Those limitations preclude causal inference; rather, our aim was to identify major predictors of fatal outcomes in pediatric RMSF and improve our understanding of its clinical and epidemiologic profile.

In summary, RMSF remains a major cause of severe pediatric illness and death in Sonora. Further research is needed to elucidate host, pathogen, and environmental determinants of RMSF-related mortality and long-term sequelae, especially among low-income communities, children >10 years, and Indigenous populations. Continued investment in diagnostic testing, education, and prevention is essential for reducing the burden of this treatable disease.

Appendix Additional information on predictors of fatal outcomes among pediatric patients hospitalized for Rocky Mountain spotted fever, Sonora, Mexico, 2004–2024.
