# Supplementary material for: Predictors of Fatal Outcomes among Pediatric Patients Hospitalized for Rocky Mountain Spotted Fever, Sonora, Mexico, 2004–2024
Source: Emerg Infect Dis. 2026 Feb;32(2):180–90. doi: 10.3201/eid3202.251223 (PMC12928218; doi:10.3201/eid3202.251223)
Supplement: Appendix — Additional information on predictors of fatal outcomes among pediatric patients hospitalized for Rocky Mountain spotted fever, Sonora, Mexico, 2004–2024. [file 25-1223-Techapp-s1.pdf]

Article DOI: https://doi.org/10.3201/eid3202.251223

*EID cannot ensure accessibility for supplementary materials supplied by authors. Readers who have difficulty accessing supplementary content should contact the authors for assistance.*

# Predictors of Fatal Outcomes among Pediatric Patients Hospitalized for Rocky Mountain Spotted Fever, Sonora, Mexico, 2004–2024

## Appendix

**Appendix Table 1.** Clinical features, vital signs, and complications during hospital admission for children with Rocky Mountain spotted fever, Sonora Mexico (2004 - 2024)

| Characteristic | No. cases | Fatal cases | Nonfatal cases | Total | p value† |
|---|---|---|---|---|---|
| Median no. days from symptom onset to admission (IQR) | 500 | 6.0 (4.0–7.0) | 5.0 (3.0–7.0) | 5.0 (3.0–7.0) | **0.001**‡ |
| Signs and symptoms on admission | | | | | |
| Fever | 500 | 99 (100) | 401 (100) | 500 (100) | NA |
| Malaise | 465 | | | | |
| Before onset of fever | | 20 (22) | 61 (16) | 81 (17) | 0.32 |
| After onset of fever | | 31 (34) | 153 (41) | 184 (40) | |
| Began simultaneously with fever | | 41 (45) | 159 (43) | 200 (43) | |
| Headache | 500 | 88 (89) | 350 (87) | 438 (88) | 0.74 |
| Vomiting | 500 | 69 (70) | 200 (50) | 269 (54) | **<0.001** |
| Arthralgia | 500 | 77 (78) | 282 (70) | 359 (72) | 0.17 |
| Myalgia | 499 | 81 (83) | 302 (75) | 383 (77) | 0.14 |
| Conjunctivitis | 499 | 14 (14) | 47 (12) | 61 (12) | 0.5 |
| Diarrhea | 500 | 59 (60) | 181 (45) | 240 (48) | 0.01 |
| Exanthema | 500 | 98 (99) | 379 (95) | 477 (95) | 0.06 |
| On feet | 499 | 87 (88) | 297 (74) | 484 (77) | 0.003 |
| On palms | 500 | 89 (90) | 315 (79) | 404 (81) | |
| Petechial rash | 500 | 88 (89) | 293 (73) | 381 (76) | **<0.001** |
| Hemorrhage (from any site) | 492 | 65 (68) | 97 (24) | 162 (33) | **<0.001** |
| From gingival tissues | 492 | 19 (20) | 24 (6.1) | 43 (8.7) | **<0.001** |
| Epistaxis | 492 | 23 (24) | 46 (12) | 69 (14) | 0.003 |
| Hematemesis | 492 | 23 (24) | 19 (4.8) | 42 (8.5) | **<0.001** |
| Melena | 492 | 13 (14) | 10 (2.5) | 23 (4.7) | **<0.001** |
| From genitourinary tract | 492 | 14 (15) | 14 (3.5) | 28 (5.7) | **<0.001** |
| Other | 492 | 22 (23) | 14 (3.5) | 36 (7.3) | **<0.001** |
| Intubation on admission | 500 | 65 (66) | 67 (17) | 132 (26) | **<0.001** |
| Respiratory distress | 500 | 39 (39) | 39 (9.7) | 78 (16) | **<0.001** |
| Mental status on admission | 499 | | | | |
| No alteration | | 23 (23) | 285 (71) | 308 (62) | **<0.001** |
| Confused/disoriented/lethargic | | 71 (72) | 112 (28) | 183 (37) | |
| Seizing | | 5 (5.1) | 3 (0.8) | 8 (1.6) | |
| Vital signs | | | | | |
| Median temperature, °C (IQR) | 465 | 37.4 (36.3–38.2) | 37.6 (36.9–38.4) | 37.5 (36.8–38.4) | 0.04 |
| Median heart rate, bpm (IQR) | 472 | 120 (106–142) | 120 (105–138) | 120 (105–140) | 0.48 |
| Median respiratory rate, rpm (IQR) | 477 | 28 (24–38) | 26 (22–32) | 26 (22–32) | 0.007 |
| Median oxygen saturation, % (IQR) | 483 | 96.0 (86.5–98.0) | 97.0 (96.0–98.0) | 97.0 (95.0–98.0) | **<0.001** |
| Edema | 500 | 75 (76) | 202 (50) | 277 (55) | **<0.001** |
| Eye edema | 500 | 65 (66) | 134 (33) | 199 (40) | **<0.001** |
| Wrist edema | 500 | 63 (64) | 143 (36) | 206 (41) | **<0.001** |
| Ankle edema | 500 | 67 (68) | 162 (40) | 229 (46) | **<0.001** |
| Abdominal pain | 500 | 62 (63) | 222 (55) | 284 (57) | 0.21 |
| Hepatomegaly | 500 | 55 (56) | 131 (33) | 186 (37) | **<0.001** |

| Characteristic | No. cases | Fatal cases | Nonfatal cases | Total | p value† |
|---|---|---|---|---|---|
| Ecchymosis | 498 | 53 (54) | 60 (15) | 113 (23) | **<0.001** |
| Confusion | 499 | 76 (78) | 134 (33) | 210 (42) | **<0.001** |
| Seizures | 500 | 36 (36) | 26 (6.5) | 62 (12) | **<0.001** |
| Encephalitis | 500 | 20 (20) | 22 (5.5) | 42 (8.4) | **<0.001** |
| Coma | 500 | 24 (24) | 14 (3.5) | 38 (7.6) | **<0.001** |
| Pneumonia | 500 | 13 (13) | 43 (11) | 56 (11) | 0.48 |
| Pulmonary edema | 500 | 18 (18) | 26 (6.5) | 44 (8.8) | **<0.001** |
| Hypovolemia | 500 | 51 (52) | 72 (18) | 123 (25) | **<0.001** |
| Shock | 500 | 79 (80) | 135 (34) | 214 (43) | **<0.001** |
| Hypotensive | 500 | 41 (41) | 48 (12) | 89 (18) | **<0.001** |
| Septic | | 26 (26) | 85 (21) | 111 (22) | |
| Mixed | | 12 (12) | 2 (0.5) | 14 (2.8) | |

*Values are no. (%) except as indicated. Bold font indicates statistical significance by Bonferroni correction (p<0.001) except as indicated. bpm, beats per minute; rpm, respirations per minute.
†Based on Fisher exact test, Pearson Chi-Square or Kruskal Wallis ANOVA.
‡Statistically significant (p<0.05)

**Appendix Table 2**. Abnormal laboratory findings at time of hospitalization in hospitalized children with Rocky Mountain spotted fever, Sonora, Mexico

| Indicator | No. cases | Fatal cases | Nonfatal cases | Total | p value† |
|---|---|---|---|---|---|
| Abnormal hemoglobin | 497 | 39 (41) | 150 (37) | 189 (38) | 0.561 |
| Abnormal hematocrit | 494 | 61 (64) | 242 (61) | 303 (61) | 0.559 |
| Abnormal leukocytes | 498 | 71 (73) | 179 (45) | 250 (50) | **<0.001** |
| Abnormal lymphocytes | 475 | 70 (78) | 221 (57) | 291 (61) | **<0.001** |
| Abnormal neutrophils | 477 | 72 (77) | 143 (37) | 215 (45) | **<0.001** |
| Abnormal neutrophil-to-lymphocyte ratio, >6 | 471 | 55 (63) | 157 (41) | 212 (45) | **<0.001** |
| Abnormal platelets | 497 | 95 (98) | 326 (82) | 421 (85) | **<0.001** |
| Abnormal serum procalcitonin | 337 | 71 (100) | 258 (97) | 329 (98) | 0.212 |
| Abnormal prothrombin time | 488 | 93 (97) | 322 (82) | 415 (85) | **<0.001** |
| Abnormal partial thromboplastin time | 486 | 86 (90) | 243 (62) | 329 (68) | **<0.001** |
| Abnormal aspartate aminotransferase | 488 | 93 (99) | 354 (90) | 447 (92) | 0.002 |
| Abnormal alanine aminotransferase | 487 | 91 (97) | 305 (78) | 396 (81) | **<0.001** |
| Abnormal lactate dehydrogenase | 384 | 77 (96) | 272 (89) | 349 (91) | 0.079 |
| Abnormal serum sodium level | 492 | 79 (81) | 320 (81) | 399 (81) | >0.99 |
| Abnormal serum creatinine | 451 | 79 (88) | 161 (45) | 240 (53) | **<0.001** |
| Abnormal serum albumin | 416 | 72 (91) | 226 (67) | 298 (72) | **<0.001** |
| Abnormal serum total protein | 417 | 63 (81) | 200 (59) | 263 (63) | **<0.001** |

*Values are no. (%). Bold font indicates statistical significance by Bonferroni correction (p<0.001)
†Based on Fisher exact test or Kruskal-Wallis ANOVA.

**Appendix Table 3.** Clinical features, vital signs, and complications during hospitalization in children that survived Rocky Mountain spotted fever infection, Sonora Mexico (2004 - 2024)

| Characteristics | No. case-patients | Discharged with sequalae, n = 64 | Discharged at baseline health, n = 336 | p value† |
|---|---|---|---|---|
| # days from symptom onset to initial presentation for care (IQR) | 400 | 6.0 (4.0–7.5) | 5.0 (3.0–6.0) | **<0.001‡** |
| # days from symptom onset to doxycycline administration (IQR) | 399 | 6.0 (4.9–7.9) | 5.0 (3.0–6.8) | **<0.001‡** |
| Signs and symptoms on admission | | | | |
| Fever | 400 | 64 (100) | 336 (100) | NA |
| Malaise | 372 | | | |
| Before onset of fever | | 7 (12) | 54 (17) | 0.54 |
| After onset of fever | | 27 (45) | 126 (40) | |
| Began simultaneously with fever | | 26 (43) | 132 (42) | |
| Headache | 400 | 51 (80) | 298 (89) | 0.06 |
| Vomiting | 400 | 31 (48) | 168 (50) | 0.89 |
| Arthralgia | 400 | 55 (86) | 226 (67) | 0.003 |
| Myalgia | 400 | 56 (88) | 245 (73) | 0.01 |
| Conjunctivitis | 399 | 10 (16) | 37 (11) | 0.29 |
| Diarrhea | 400 | 30 (47) | 150 (45) | 0.79 |
| Exanthema | 400 | 63 (98) | 315 (94) | 0.23 |
| On feet | 399 | 58 (91) | 238 (71) | **<0.001** |
| On palms | 400 | 60 (94) | 254 (76) | **<0.001** |
| Petechial rash | 400 | 60 (94) | 232 (69) | **<0.001** |
| Hemorrhage (from any site) | 395 | 27 (43) | 70 (21) | **<0.001** |
| From gingival tissues | 395 | 7 (11) | 17 (5) | 0.08 |
| Epistaxis | 395 | 10 (16) | 36 (11) | 0.28 |
| Hematemesis | 395 | 9 (14) | 10 (3) | **<0.001** |
| Melena | 395 | 5 (8) | 5 (2) | 0.01 |
| From genitourinary tract | 395 | 6 (10) | 8 (2) | 0.01 |
| Other | 395 | 6 (10) | 8 (2) | 0.01 |
| Clinical features | | | | |
| Intubation on admission | 400 | 35 (55) | 32 (10) | **<0.001** |
| Respiratory distress | 400 | 17 (27) | 22 (7) | **<0.001** |
| Temperature, ˚C (IQR) | 378 | 37.6 (36.8–38.6) | 37.6 (36.9–38.4) | 0.94 |
| Heart rate, bpm (IQR) | 384 | 124 (110–140) | 120 (104–138) | 0.05 |
| Respiratory rate, rpm (IQR) | 384 | 28 (22–36) | 25 (22–30) | 0.02 |
| Oxygen saturation, % (IQR) | 390 | 97.0 (93.0–98.0) | 98.0 (96.0–98.0) | 0.006 |
| Edema | 400 | 58 (91) | 144 (43) | **<0.001** |
| Eye edema | 400 | 48 (75) | 86 (26) | **<0.001** |
| Wrist edema | 400 | 52 (81) | 91 (27) | **<0.001** |
| Ankle edema | 400 | 51 (80) | 111 (33) | **<0.001** |
| Abdominal pain | 400 | 47 (73) | 174 (52) | 0.002 |
| Hepatomegaly | 400 | 39 (61) | 92 (27) | **<0.001** |
| Ecchymosis | 398 | 27 (42) | 33 (10) | **<0.001** |
| Confusion | 400 | 46 (72) | 88 (26) | **<0.001** |
| Seizures | 400 | 13 (20) | 13 (4) | **<0.001** |
| Encephalitis | 400 | 15 (23) | 7 (2) | **<0.001** |
| Coma | 400 | 7 (11) | 7 (2) | 0.003 |
| Pneumonia | 400 | 25 (39) | 18 (5) | **<0.001** |
| Pulmonary edema | 400 | 11 (17) | 15 (5) | **<0.001** |
| Hypovolemia | 400 | 32 (50) | 40 (12) | **<0.001** |
| Shock | 400 | 46 (72) | 89 (26) | **<0.001** |

*Values are no. (%) except as indicated. Bold font indicates statistical significance by Bonferroni correction (p<0.001) except as indicated. bpm, beats per minute; IQR, interquartile range; rpm, respirations per minute.
†Based on Fisher exact test, Pearson Chi-Square or Kruskal Wallis ANOVA
‡Statistically significant (p<0.05)

**Appendix Table 4.** Laboratory findings at time of hospitalization in hospitalized children that survived infection with Rocky Mountain spotted fever, Sonora, Mexico (2004 - 2024)

| Indicator | No. patients | Discharged with sequalae, n = 64 | Discharged at baseline health, n = 336 | p value† |
|---|---|---|---|---|
| Hemoglobin, g/dL | 400 | 11.3 (9.95–12.1) | 11.6 (10.4–12.6) | 0.04 |
| Hematocrit, % | 398 | 32.5 (28.0–35.3) | 34.0 (30.8–37.1) | 0.006 |
| Blood leukocytes, × $10^3$ cells/µL | 400 | 9.3 (6.7–15.4) | 8.1 (5.7–12.1) | 0.04 |
| Absolute lymphocyte count, × $10^3$ cells/µL | 384 | 1.2 (0.6–2.0) | 1.1 (0.8–2.0) | 0.49 |
| Absolute neutrophil count, × $10^3$ cells/µL | 383 | 7.0 (5.0–12.5) | 5.7 (3.9–8.7) | 0.03 |
| Neutrophil-to-lymphocyte ratio | 383 | 5.1 (3.5–11.1) | 5.0 (2.9–8.5) | 0.18 |
| Platelets, × $10^3$ cells/µL | 399 | 22 (15–33) | 72 (35–133) | **<0.001** |
| Serum procalcitonin, ng/mL | 270 | 9 (3–17) | 4 (1–10) | 0.001 |
| Prothrombin time, sec | 391 | 15.1 (13.9–17.1) | 15.1 (13.9–16.0) | 0.76 |
| Partial thromboplastin time, sec | 389 | 40 (35–46) | 38 (32–42) | 0.02 |
| Aspartate aminotransferase, U/L | 393 | 158 (117–221) | 104 (57–165) | **<0.001** |
| Alanine aminotransferase, U/L | 392 | 68 (45–93) | 53 (31–81) | 0.002 |
| Lactate dehydrogenase, U/L | 304 | 889 (798–1211) | 709 (515–956) | **<0.001** |
| Serum sodium level, mq/L | 394 | 132 (127–135) | 133 (130–136) | 0.16 |
| Serum creatinine, mg/dL | 360 | 0.6 (0.4–1.2) | 0.5 (0.4–0.6) | 0.01 |
| Serum albumin, g/dL | 336 | 2.8 (2.6–3.0) | 3.3 (2.9–4.0) | **<0.001** |
| Serum total protein, mg/dL | 338 | 5.2 (4.7–5.5) | 5.9 (5.2–6.5) | **<0.001** |

*Values are no. (interquartile range) except as indicated. Bold font indicates statistical significance by Bonferroni correction (p<0.001).
†Based on Fisher exact test or Kruskal Wallis ANOVA

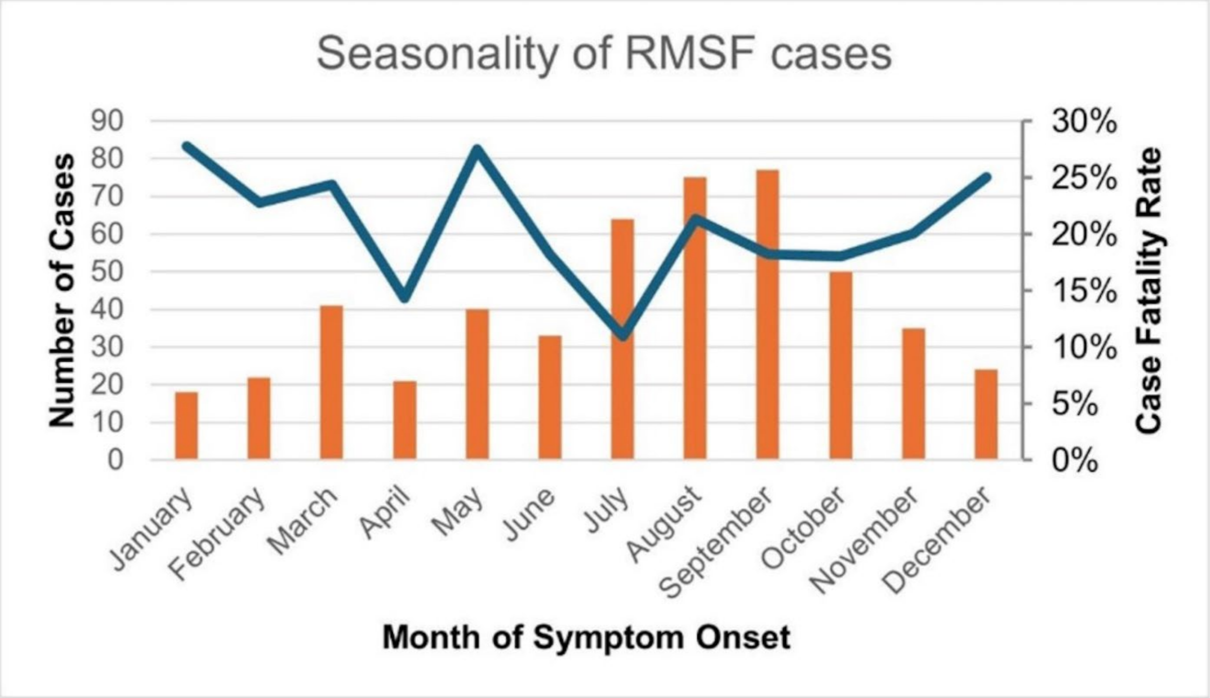


**Appendix Figure**. Seasonality of RMSF infections in the Sonora pediatric population served by HIES (2004–2024).
